# Effectiveness and Safety of Rituximab in Patients with Refractory Juvenile Idiopathic Arthritis: A Tertiary Center Experience

**DOI:** 10.3390/ph19091329

**Published:** 2026-08-23

**Authors:** Ekaterina I. Alexeeva, Natalia M. Kondrateva, Tatyana M. Dvoryakovskaya, Dmitry A. Kudlay, Ivan A. Kriulin, Maria S. Botova, Irina T. Tsulukiya, Elizaveta A. Krekhova, Meiri S. Shingarova, Maria Y. Kokina, Anna N. Fetisova, Tatiana Y. Kriulina, Kseniya B. Isaeva, Aleksandra M. Chomakhidze, Christina V. Chibisova, Mikhail M. Kostik

**Affiliations:** 1 Department of Pediatric Rheumatology, National Medical Research Center for Children’s Health, 119296 Moscow, Russia; alekatya@yandex.ru (E.I.A.); 131nk@mail.ru (N.M.K.); tbzarova@mail.ru (T.M.D.); 9671819676@yandex.ru (I.A.K.); mariabotova22@gmail.com (M.S.B.); irinatsulukiya@gmail.com (I.T.T.); lizakrek@mail.ru (E.A.K.); mshingarova@mail.ru (M.S.S.); kokinamariah@yandex.ru (M.Y.K.); anna_534@mail.ru (A.N.F.); t.sinelnikova@aspirre-russia.ru (T.Y.K.); isaeva-kseniya@yandex.ru (K.B.I.); achomakhidze@mail.ru (A.M.C.); chibisova.kri@yandex.ru (C.V.C.); 2 Clinical Institute of Children’s Health Named After N.F. Filatov, Department of Pediatrics and Pediatric Rheumatology, I.M. Sechenov First Moscow State Medical University (Sechenov University), 119435 Moscow, Russia; 3 Association of Pediatric Rheumatologists, 107078 Moscow, Russia; 4 Institute of Pharmacy, I.M. Sechenov First Moscow State Medical University (Sechenov University), 119435 Moscow, Russia; d624254@gmail.com; 5 Faculty of Bioengineering and Bioinformatics, Lomonosov Moscow State University, 119991 Moscow, Russia; 6 Department of Hospital Pediatrics, Saint Petersburg State Pediatric Medical University, Litovskaya 2, 194100 Saint Petersburg, Russia

**Keywords:** rituximab, B-cell, juvenile idiopathic arthritis, biologics, refractory course, remission

## Abstract

**Introduction:** Despite the growing spectrum of modern drugs for the treatment of juvenile idiopathic arthritis (JIA), patients with a refractory, progressive course of the disease remain a clinical challenge. B-cell-depleting therapy has demonstrated efficacy in adult rheumatoid arthritis and several pediatric rheumatic diseases, encouraging us to evaluate rituximab (RTX) for children with refractory JIA. **Objective:** This study aimed to assess the long-term efficacy and safety of rituximab therapy in children with refractory juvenile idiopathic arthritis. **Methods:** This retrospective, single-center, observational study included 106 patients with JIA who had failed previous therapy. The efficacy and safety of the therapy were evaluated at baseline and at 3, 6, 12, 24, 36, and 48 months post-initial rituximab infusion using the American College of Rheumatology Pediatric Criteria (ACRpedi 30/50/70/90), C. Wallace criteria for inactive disease/remission, and the JADAS71 activity index until patient completion or withdrawal from the study. The primary endpoint was achieving an ACRpedi50 response by 3 months, and the secondary endpoint was achieving inactive disease status according to C. Wallace criteria by 6 months of follow-up. **Results:** An ACRpedi50 response was achieved in 78/100 (78%) patients by month 3 of rituximab therapy, and inactive disease status was reached in 48/93 (52%) patients by month 6. More than half of the patients demonstrate inactive disease or remission at each observation point. During rituximab therapy, oral glucocorticoids were discontinued in 18/57 (32%) patients, and 28/57 (49%) patients were able to halve their corticosteroid dose (*p* = 0.03). Rituximab demonstrated an acceptable safety profile, with a total of 351 adverse events (non-serious AEs: 304/351, 87%) and a serious AE rate of 0.8 per 100 patient-years. **Conclusions:** Rituximab can reduce disease activity and lower the dose of oral glucocorticoids in patients with refractory JIA, serving as a viable therapeutic option with an acceptable safety profile.

## 1. Introduction

Juvenile idiopathic arthritis (JIA) is the most common chronic rheumatic disease of childhood. JIA encompasses all forms of arthritis of unknown etiology with onset before 16 years of age and a duration exceeding 6 weeks [1]. According to the International League of Associations for Rheumatology (ILAR) classification, JIA is categorized into several distinct subtypes: systemic-onset JIA (sJIA), oligoarticular JIA, polyarticular JIA (RF-negative), polyarticular JIA (RF-positive), enthesitis-related arthritis, psoriatic arthritis, and undifferentiated arthritis [1]. The most severe JIA subtypes are systemic arthritis and polyarticular JIA (RF-positive), which, without adequate treatment, can lead to irreversible joint damage or other serious complications, including pulmonary involvement, amyloidosis, macrophage activation syndrome, and death [2,3].

The goal of JIA therapy is to control disease symptoms, prevent joint destruction and growth impairment, and achieve a good quality of life and social integration [4]. In 2011, the “treat-to-target” principle was formulated, establishing key primary outcomes as a 50% improvement according to Pediatric American College of Rheumatology criteria (ACRpedi50) within 3 months, inactive disease status per C. Wallace criteria after 6 months of therapy, and fever resolution within 1 week for patients with sJIA [4]. First-line therapy for non-systemic JIA variants includes nonsteroidal anti-inflammatory drugs (NSAIDs), intra-articular glucocorticoid administration, and conventional synthetic disease-modifying antirheumatic drugs (DMARDs). Second-line therapy includes biologic DMARDs with different mechanisms of action, such as tumor necrosis factor (TNF) inhibitors, T-lymphocyte co-stimulation blockers, and interleukin-6 inhibitors [5]. Interleukin-1 inhibitors (anakinra and canakinumab) and interleukin-6 receptor antagonists (tocilizumab) are used as a first-line biologic treatment for systemic JIA [5,6,7]. According to the ACR guidelines, RTX may be considered as last-line treatment of non-systemic types of JIA after DMARDs, TNF- α inhibitors, anti-IL 6 and abatacept. For sJIA, RTX is considered a last-ditch effort treatment after DMARDs, anti-IL6 and anti-IL1 therapy [5]. Despite the available highly effective treatment options, 15–30% of patients exhibit persistent disease activity [7,8].

B cells participate in several immune mechanisms, including autoantibody production, antigen processing and presentation, secretion of pro-inflammatory cytokines, T-lymphocyte stimulation, and maintenance of immunological memory [9].

Rituximab (RTX) is a chimeric monoclonal antibody targeting CD20-expressing B lymphocytes. It induces antibody-dependent cellular cytotoxicity and complement-mediated B-cell lysis. In 1997, RTX was approved for the treatment of B-cell non-Hodgkin lymphoma [10]. The first clinical trials demonstrating the efficacy of rituximab in patients with rheumatoid arthritis were conducted in 1998, and in 2006, the U.S. Food and Drug Administration (FDA) approved RTX for the treatment of adult rheumatoid arthritis [11]. Currently, in pediatric rheumatology, RTX is successfully used to treat ANCA-associated vasculitis, systemic lupus erythematosus, systemic sclerosis, IgG4-related disease, nephrotic syndrome, and immune thrombocytopenic purpura [12,13,14]. Numerous studies on the efficacy and safety of RTX have been conducted in adult patients with rheumatoid arthritis [11,15,16]. Several case reports and cohort studies evaluating rituximab in children with JIA have been published [17,18,19]. The first large study evaluating the efficacy and safety of RTX in children with JIA was published in 2011. It included 55 patients and had a follow-up duration of 96 weeks [17]. Our study aims to evaluate the efficacy and safety of RTX in children with refractory JIA.

## 2. Results

### 2.1. Demographic Characteristics and Prior Therapy

The study included 106 patients (32 boys, 74 girls). The median age at disease onset was 3.9 years (IQR 2.3–8.8), and the median disease duration prior to RTX initiation was 3.8 years (IQR 1.5–7.2). The cohort included 6/106 (5.6%) patients with RF-negative polyarthritis, 2/106 (1.9%) with persistent oligoarthritis, 2/106 (1.9%) with extended oligoarthritis, 3/106 (2.8%) with enthesitis-related arthritis, and 24/106 (22.6%) with RF-positive polyarthritis. The largest group consisted of children diagnosed with systemic JIA (sJIA): 69/106 (65.1%). A history of uveitis was observed in 7/106 (6.6%) patients, and a history of macrophage activation syndrome (MAS) was noted in 26/106 (24.5%) patients—all of whom had sJIA (26/69, 37.7%) (Table 1).

Prior to RTX initiation, all patients had received conventional or targeted synthetic disease-modifying antirheumatic drugs (DMARDs): 99/106 (93%) received methotrexate, 22/106 (21%) received leflunomide, 62/106 (59%) received cyclosporine A, 1/106 (1%) received mycophenolate mofetil, 1/106 (1%) received upadacitinib, and 4/106 (4%) received cyclophosphamide. Prior to RTX, combination therapy was administered to 55/106 (52%) patients: 53/106 (50%) received methotrexate and cyclosporine, 1/106 (1%) received cyclophosphamide and methotrexate, and 1/106 (1%) received leflunomide and cyclosporine.

Prior to RTX initiation, 61/106 (58%) patients had been treated with biological DMARDs: 39/106 (37%) with one biological DMARD, 14/106 (13%) with two, 5/106 (5%) with three, 2/106 (2%) with four, and 1/106 (1%) patient with five biological DMARDs. Among these, 9/106 (9%) patients with active systemic JIA received canakinumab and tocilizumab as first-line biologic agents. A total of 45/106 (45%) patients were biologically naïve (having received no prior biologic DMARDs) (Table 1).

### 2.2. Disease Activity and Concomitant Treatment at Baseline (RTX Initiation)

High disease activity was present at baseline: the median count of active joints was eight (IQR 4–16), the median count of joints with limited range of motion was eight (IQR 4–18), and the median morning stiffness duration was 30 min (IQR 0–60) (Table 2). Extra-articular manifestations included active uveitis in 3/106 (3%) patients, Sjögren’s syndrome in 2/106 (2%) patients, and systemic manifestations in 62/106 (58%) children (with sJIA): 19/106 (18%) had fever, 58/106 (55%) had rash, 25/106 (24%) had lymphadenopathy, 42/106 (40%) had hepatomegaly and/or splenomegaly, and 9/106 (8%) had serositis. In 6/106 (6%) patients, RTX was initiated due to high disease activity complicated by macrophage activation syndrome (Table 2).

Based on CHAQ scores, the functional disability index indicated moderate impairment, with a median score of 1.6 (IQR 1.1–1.9). Physician-reported disease activity (MDVAS) was high, with a median score of 42 mm (IQR 35–46). Parent/patient global assessment (pVAS) indicated poor health status, with a median score of 57 mm (IQR 48–64). The median JADAS71 score was 21 (IQR 14.8–30.1). Laboratory parameters reflected marked inflammatory activity: the median ESR was 43 mm/h (IQR 25–56), the median serum C-reactive protein (CRP) level was 34.5 mg/L (IQR 11.8–55), and the median hemoglobin level was 103 g/L (IQR 89–115). Patients with RF-positive polyarthritis exhibited high immunological activity: the median anti-CCP level was 138 IU/L (IQR 11–431), and the median RF level was 157 IU/L (IQR 88–263) (Table 2).

At RTX initiation, 57/106 (54%) patients were receiving oral glucocorticoids (GCs), 41/106 (39%) intravenous GCs, 8/106 (8%) intra-articular GC injections, 75/106 (71%) methotrexate, 15/106 (14%) leflunomide, 62/106 (58%) cyclosporine A, 1/106 (1%) mycophenolate mofetil, and 1/106 (1%) upadacitinib. Despite ongoing treatment, disease activity could not be controlled. Combination therapy was administered to 51/106 (48%) patients: 11/106 (10%) received oral GCs and methotrexate, 27/106 (25%) received oral GCs + cyclosporine + methotrexate, 10/106 (9%) received cyclosporine and methotrexate, and 3/106 (3%) received oral GCs + leflunomide.

### 2.3. Efficacy of Rituximab Therapy

Treatment efficacy was evaluated at 3, 6, 12, 24, and 48 months following the first RTX infusion. The evaluation included the following parameters: number of joints with limited range of motion, number of active joints, presence of systemic manifestations, morning stiffness duration, physician global assessment of disease activity (visual analogue scale, VAS), parent/patient global assessment (visual analogue scale, VAS), Childhood Health Assessment Questionnaire disability index (CHAQ), and 71-joint Juvenile Arthritis Disease Activity Score (JADAS71).

According to the “treat-to-target” approach, a 50% improvement according to ACRpedi criteria (ACRpedi50) at 3 months following the first RTX infusion was achieved by 78/100 (78%) patients. Inactive disease status according to C. Wallace criteria at 6 months was achieved by 48/93 (52%) patients.

**Figure 1:**
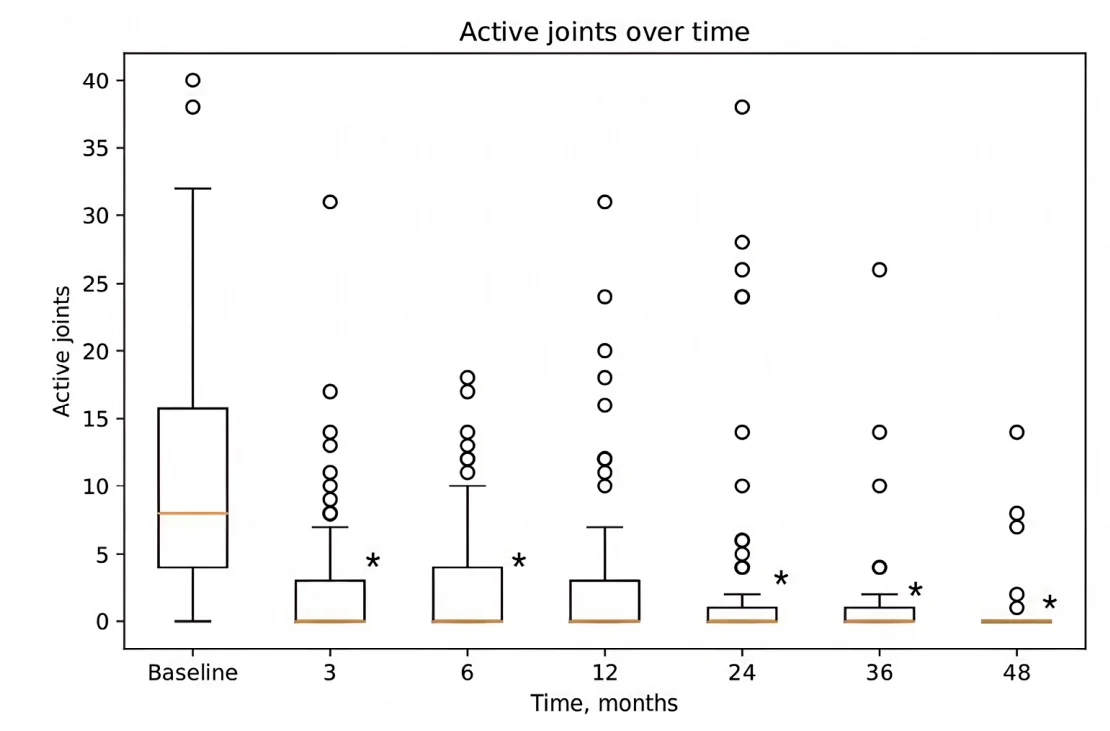
Active joint count during RTX treatment (* *p* < 0.05 compared to baseline).

**Figure 2:**
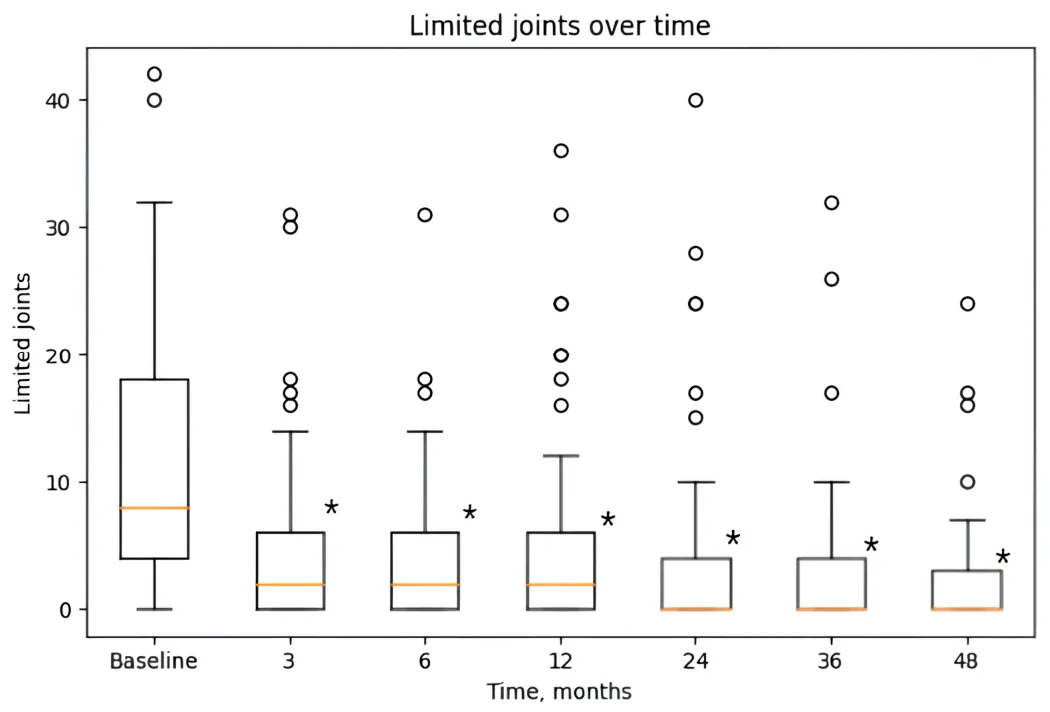
Joints with limited range of motion during RTX treatment (* *p* < 0.05 compared to baseline).

Three months after RTX initiation, a significant reduction in arthritis activity was observed: the median number of joints with active arthritis decreased from eight (IQR 4–18) to two (IQR 0–6) (p=0.001); the median number of joints with limited range of motion decreased from eight (IQR 4–16) to zero (IQR 0–3) (p=0.001); and the median morning stiffness duration decreased from 30 min (IQR 0–60) to 0 min (IQR 0–0) (p=0.001) (Table 3, Figure 1 and Figure 2).

Throughout the follow-up period, the median number of joints with active arthritis remained at zero (p<0.05), and morning stiffness was absent (p<0.05) (Table 3). The median number of joints with limited range of motion decreased significantly compared to baseline, reaching zero by year 2 of follow-up (p<0.05) (Table 3).

Three months after RTX initiation, a significant improvement in the functional ability of JIA patients was observed: the CHAQ disability index decreased from 1.6 (IQR 1.1–1.9) to 0.08 (IQR 0.02–0.40) (p<0.05), indicating minimal to no functional impairment.

Physician-assessed disease activity (MDVAS) decreased from 42 mm (IQR 35–46) to 12 mm (IQR 7–21) (p<0.05). Parent/patient global assessment of overall well-being (pVAS) improved, with scores decreasing from 57 mm (IQR 48–64) to 18 mm (IQR 9–34) (p<0.05). Disease activity, as measured by the JADAS71 score, decreased from 21 (IQR 14.8–30.1) to 2.7 (IQR 0–8.9) (p=0.003).

Throughout the follow-up period, the improvement in the CHAQ disability index was sustained. Physician-assessed disease activity (MDVAS) and parent/patient global assessment of overall well-being (pVAS) remained low (p<0.05). Disease activity, measured by the JADAS71 score, decreased further, reaching zero (IQR 0–5; p=0.006) by month 24 of follow-up (Table 3).

### 2.4. Efficacy of Rituximab Therapy According to ACRpedi Criteria and C. Wallace Criteria for Inactive Disease/Remission

**Figure 3:**
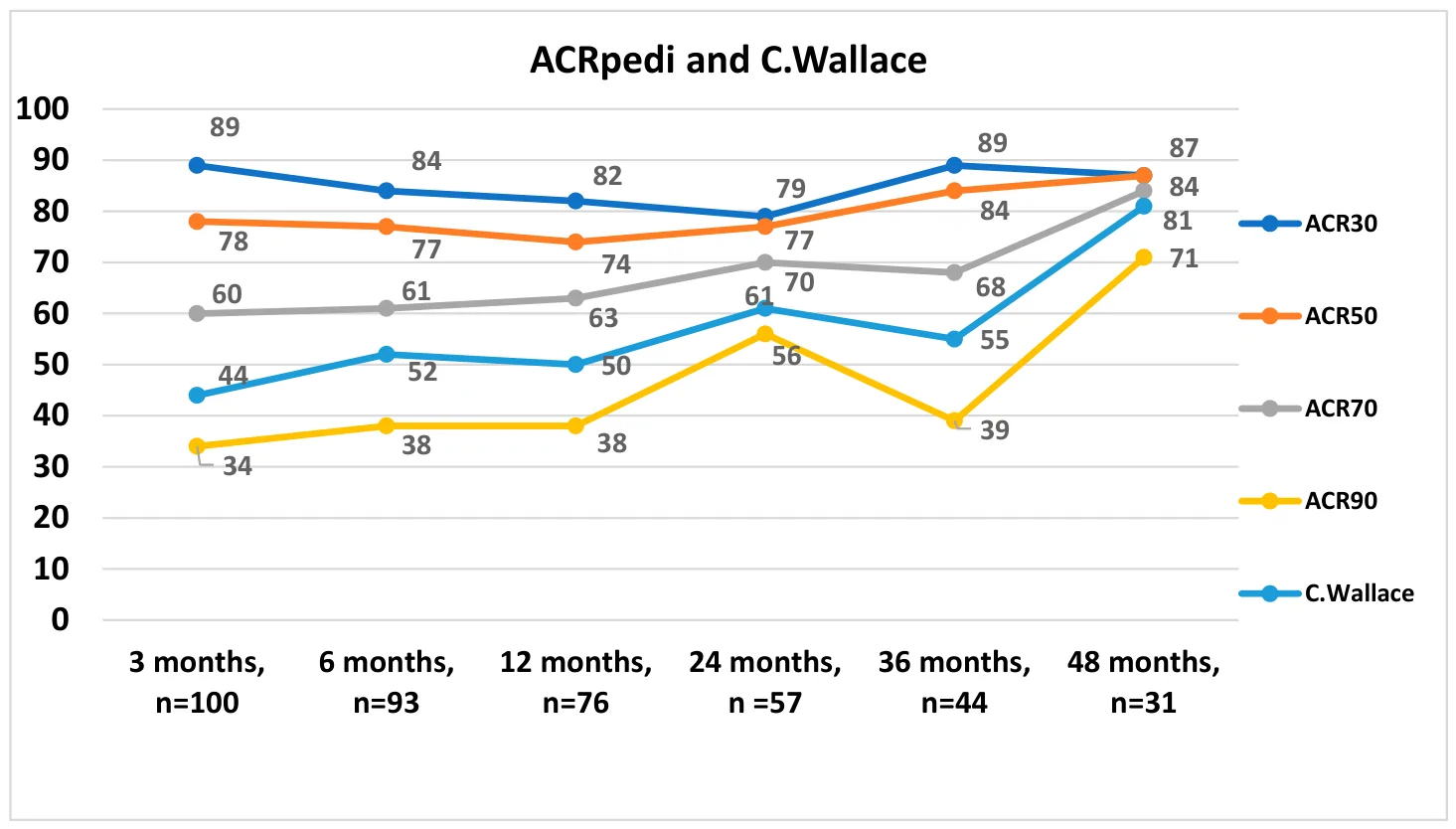
Efficacy of rituximab according to ACRpedi and C. Wallace criteria.

At the 3-month follow-up, an ACRpedi30 response was achieved in 89/100 (89%) patients, and the primary endpoint (ACRpedi50) was achieved in 78/100 (78%) patients. ACRpedi70 response was observed in 60/100 (60%) patients, and ACRpedi90 response in 34/100 (34%) patients. Inactive disease according to C. Wallace criteria was achieved in 44/100 (44%) patients (Figure 3). At the 6-month follow-up, ACRpedi30/50/70/90 response was achieved in 78/93 (84%), 72/93 (77%), 57/93 (61%), and 35/93 (38%) patients, respectively. Inactive disease according to C. Wallace criteria was achieved in 48/93 (52%) patients (Figure 3).

At the 1-year follow-up, ACRpedi30/50/70/90 responses were achieved in 62/76 (82%), 56/76 (74%), 48/76 (63%), and 29/76 (38%) patients, respectively. Inactive disease/remission according to C. Wallace criteria was achieved in 38/76 (50%) patients (Figure 3).

At the 2-year follow-up, ACRpedi30/50/70/90 responses were achieved in 45/57 (79%), 44/57 (77%), 40/57 (70%), and 32/57 (56%) patients, respectively. Inactive disease according to C. Wallace criteria was achieved in 35/57 (61%) patients (Figure 3).

At the 3-year follow-up, ACRpedi30/50/70/90 responses were observed in 39/44 (89%), 37/44 (84%), 30/44 (68%), and 17/44 (39%) patients, respectively. At the 4-year follow-up, ACRpedi30/50/70/90 responses were observed in 27/31 (87%), 27/31 (87%), 26/31 (84%), and 22/31 (71%) patients, respectively. Remission status according to C. Wallace criteria was achieved in 24/44 (55%) patients at 3 years of follow-up and in 25/31 (81%) patients at 4 years of follow-up.

**Figure 4:**
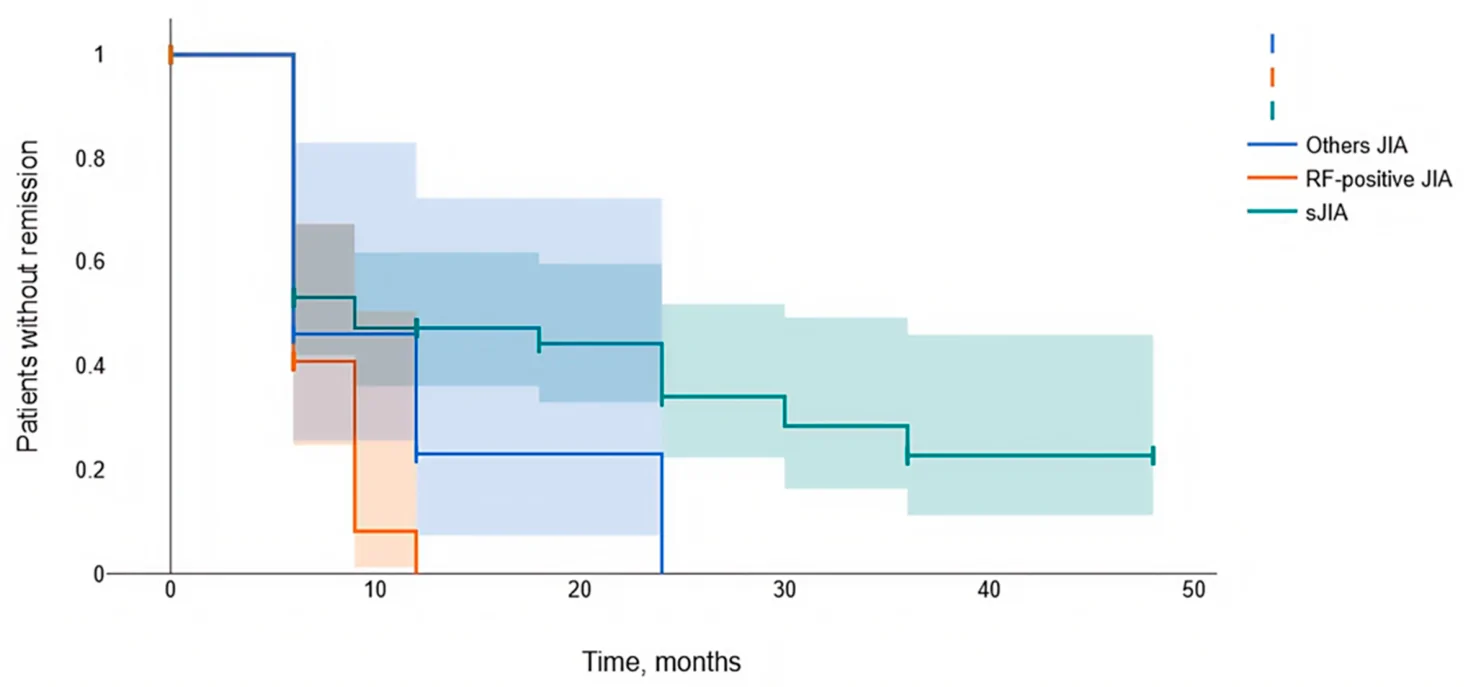
Inactive disease achievement (sJIA, polyarticular FF-positive JIA and other JIA groups).

Approximately half of the patients achieved the inactive stage of the disease for the first time within the first 6 months of therapy. Inactive disease achievement (Figure 4) was recorded most rapidly in the non-systemic and non-sJIA group of patients (mostly at the first 6 months of follow-up, *p* = 0.08). In the RF-positive polyarthritis cohort, the achievement of inactive disease was also recorded during the first year of treatment (*p* = 0.012). However, several patients in this group dropped out before the end of the first year of follow-up was recorded. In the sJIA group, inactive disease was observed in approximately half of the patients during the first six months of follow-up; however, significant improvement was not achieved as rapidly thereafter (Figure 4).

### 2.5. Changes in Laboratory Parameters of JIA Activity During Rituximab Therapy

At baseline (RTX initiation), the median ESR was 43 mm/h (IQR 25–56), and the median CRP level was 34.5 mg/L (IQR 11.8–55). After 3 months, these values decreased significantly to within normal limits and remained within the reference range throughout the follow-up period (p<0.05). The median hemoglobin level increased from 103 g/L (IQR 89–115) at baseline to 120 g/L (IQR 103–127) by month 12 (p<0.05) (Table 3).

### 2.6. B-Lymphocyte Depletion

Three months after the first rituximab infusion, B-cell depletion was achieved in 95/100 (95%) patients. At 6 months, sustained depletion was observed in 75/93 (81%) patients. Depletion of CD20+ B lymphocytes (defined as B-cell count <1% of circulating lymphocytes) following the first RTX administration lasted a mean of 6.3 ± 1.5 months. By month 12, a trend toward B-cell repopulation was noted, with complete depletion persisting in 36/76 (47%) patients. At 2, 3, and 4 years post-initial infusion, complete depletion was maintained in approximately one-third of patients: 22/57 (39%), 17/44 (39%), and 8/31 (31%), respectively (Table 3).

### 2.7. Changes in Systemic Manifestations During Rituximab Therapy

**Figure 5:**
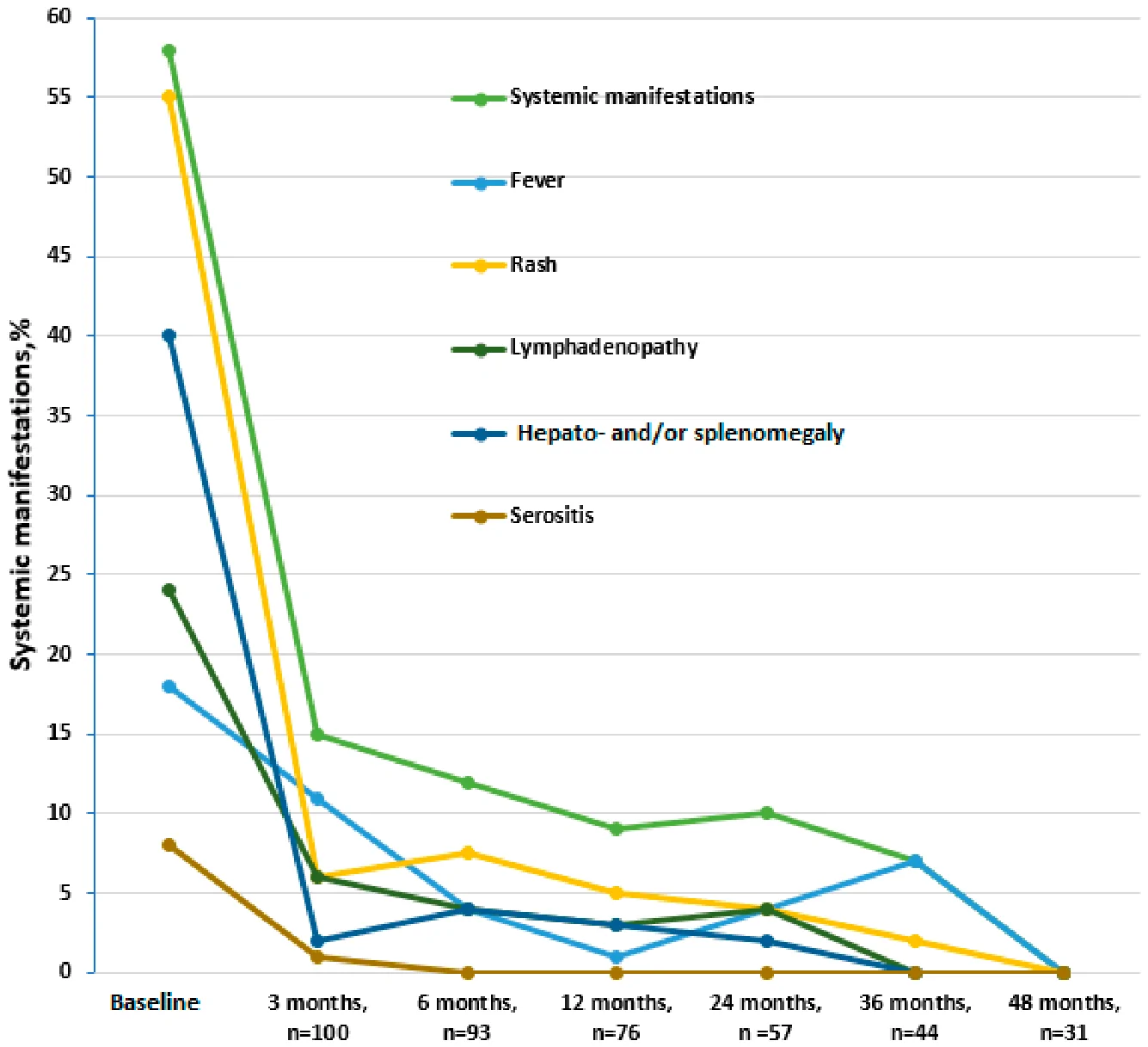
Changes in systemic features in patients with systemic juvenile idiopathic arthritis during rituximab therapy.

Prior to therapy initiation, active systemic manifestations of juvenile arthritis were observed in 62/106 (58%) patients: fever in 19/106 (19%), rash in 58/106 (55%), lymphadenopathy in 25/106 (24%), hepatomegaly and/or splenomegaly in 42/106 (40%), and serositis in 9/106 (8%). The mean number of systemic manifestations per patient was 2.22 ± 1.2 (Figure 5). At 3 months after the first rituximab infusion, the mean number of systemic manifestations per patient significantly decreased to 0.39 ± 0.8 (p<0.05). Systemic manifestations were observed in 15/100 (15%) patients: fever in 11/100 (11%), rash in 6/100 (6%), lymphadenopathy in 6/100 (6%), hepatomegaly and/or splenomegaly in 2/100 (2%), and serositis in 1/100 (1%).

A trend toward the regression of systemic manifestations was observed over the subsequent 36 months of follow-up. Systemic manifestations persisted in 11/93 (12%) patients at 6 months, 7/93 (9%) at 12 months, 6/57 (10%) at 24 months, and 3/44 (7%) at 36 months. The mean number of systemic manifestations per patient decreased throughout the follow-up period, reaching 0.31 ± 0.69 at 6 months, and 0.20 ± 0.49, 0.18 ± 0.65, and 0.12 ± 0.42 at 12, 24, and 36 months, respectively. At the 48-month follow-up, none of the patients who remained in the study exhibited systemic manifestations.

### 2.8. Glucocorticoid Therapy

At RTX initiation, 57/106 (54%) patients were receiving oral glucocorticoids (GCs), with a median daily prednisolone-equivalent dose of 0.36 mg/kg/day (IQR 0.19–0.73). High-dose oral GCs (>0.5 mg/kg/day) were administered to 23/106 (22%) patients. Intravenous GCs were administered to 41/106 (39%) patients, and intra-articular GC injections to 8/106 (8%) patients.

During rituximab therapy, oral GCs were discontinued in 18/57 (32%) patients. The median daily dose was reduced to 0.18 mg/kg/day (IQR 0.12–0.30) during the first year and to 0.15 mg/kg/day (IQR 0.10–0.25) during year 2 of follow-up. By year 4 of therapy, the median daily dose of oral GCs was 0.10 mg/kg/day (IQR 0.07–0.14) (p=0.03). Intra-articular GC injections were received by eight patients during the first RTX course, and six of them required repeated intra-articular injections during the first year of therapy. The most affected joints leading to serious disability and requiring IA GCs were knees (6/8 patients) and wrists (2/8 patients). Intravenous GCs were administered during the 2 years of follow-up only when a flare of the disease occurred.

### 2.9. Safety of Rituximab Therapy in Patients with JIA

Over the 4-year follow-up period, a total of 347 adverse events (AEs) occurred over 220 patient-years (PYs), resulting in an AE rate of 158 per 100 PYs. The majority of AEs (295/347, 85%) were classified as mild to moderate (grade < 3) according to Common Terminology Criteria for Adverse Events (CTCAE) v5.0. A total of 47/347 (13.5%) AEs were classified as serious (grade 3; primarily infusion reactions), and 5/347 (1.4%) were life-threatening or fatal (grades 4–5). More than half of all AEs occurred during the first year of rituximab therapy (206/347, 59%), primarily comprising infusion-related reactions (66/206, 32%). During the second year of therapy, 73/347 (21%) AEs were recorded; during the third year, 55/347 (16%); and during the fourth year, 13/347 (3.7%) (Table 4).

A total of 273 courses (870 infusions) of RTX were administered. Infusion-related reactions (IRRs) occurred during 98/870 infusions (11%). These IRRs manifested as nausea/vomiting in 10/98 (10%) cases, rash in 29/98 (30%), abdominal pain in 11/98 (11%), headache in 10/98 (10%), dyspnea in 21/98 (20%), flu-like syndrome in 52/98 (53%), sore throat in 15/98 (15%), facial and mucosal edema in 3/98 (3%), and anaphylaxis in 2/98 (2%). The majority of IRRs (81/98, 83%) were classified as mild, 16/98 (16%) were classified as severe but not life-threatening (grade 3), and one case required immediate medical intervention (Table 4).

Over the 4-year follow-up period, a total of 134 infectious adverse events were recorded (61 per 100 PY), the majority of which were non-severe (110/134, 82%); 23/134 (17%) were classified as grade 3; and 1/134 (0.7%)—a case of COVID-19 pneumonia—was fatal. The most common infectious events were pneumonia (41/134, 31%), ear, nose, and throat (ENT) infections (25/134, 19%), and urinary tract infections (21/134, 16%). Acute intestinal infections, skin and soft tissue infections, and herpesvirus infections were less frequent, occurring in 10/134 (7%), 7/134 (5%), and 8/134 (6%) of cases, respectively. Mucosal fungal infections were rare (4/134, 3%), most commonly presenting as oral candidiasis. There were 3/134 (2.2%) cases of sepsis following pneumonia, 1/134 (0.7%) cases of septic post-traumatic arthritis of the knee (purulent gonitis), and 1/134 (0.7%) cases of pulmonary tuberculosis.

The third category of adverse events comprised laboratory abnormalities (n=115, 52 per 100 PY), episodes of MAS (n=3, 1.4 per 100 PY; classified as severe AEs), and active uveitis (n=2, 0.9 per 100 PY). Among the laboratory abnormalities, only 3/115 (2.6%) were classified as severe (all three were cases of agranulocytosis with a risk of infection). Non-severe laboratory abnormalities included 2/115 (1.7%) cases of anemia (erythropenia), 1/115 (0.8%) episodes of thrombocytopenia, 1/115 (0.8%) cases of elevated blood urea levels, 11/115 (10%) episodes of elevated liver transaminases, and most frequently, hypogammaglobinemia (77/115, 67%). However, some investigators consider hypogammaglobinemia to be an indicator of RTX efficacy.

## 3. Discussion

We retrospectively analyzed the clinical experience of RTX use in children with juvenile idiopathic arthritis who had failed therapy with non-biologic DMARDs and biologic DMARDs with other mechanisms of action. RTX therapy led to a marked reduction in disease activity.

In 2009, Feito et al. were the first to report successful RTX use in a patient with systemic JIA presenting with active systemic manifestations and polyarthritis who had failed prior treatment with etanercept, methotrexate, and oral prednisolone [20]. Over an 18-month follow-up period, three courses of RTX were administered due to disease flares, enabling a reduction in disease activity and tapering of the oral glucocorticoid dose [20]. Subsequently, Narvaez et al. described a case series of successful RTX use in three patients with refractory systemic JIA. One year post-initial RTX infusion, all patients demonstrated significant clinical improvement with complete resolution of systemic manifestations [19]. An open-label study evaluating the efficacy and safety of repeated RTX courses in patients with refractory juvenile arthritis was published by our team in 2011. That study included 55 patients with a follow-up duration of 96 weeks. The primary endpoint was the achievement of an ACRpedi30 response by week 24 of therapy, which was attained by 98% of patients. By week 48, an ACRpedi50 response was achieved in 75% of patients. More than half of the cohort achieved clinical and laboratory remission during the study [17]. Kearsley-Fleet et al. published a cohort study of rituximab therapy in children with JIA (n=41), with a follow-up duration of 270 days post-initial infusion. The study demonstrated a significant reduction in disease activity, evidenced by a decrease in the number of joints with active arthritis and a lower JADAS71 score [18].

Our long-term observational study serves as an extension of our 2011 investigation. By expanding the cohort and establishing primary and secondary endpoints in accordance with the treat-to-target paradigm, this extended study demonstrates that RTX is an effective therapeutic option for children with treatment-resistant JIA, encompassing both articular subtypes and sJIA with active systemic manifestations. Our findings complement our prior observations as well as results reported by Kearsley-Fleet et al. [17,18].

The present study evaluated 106 patients with severe, refractory courses of various JIA subtypes who had failed glucocorticoids, DMARDs, and biologic DMARDs. In accordance with the treat-to-target paradigm for JIA therapy—wherein the primary milestone is an ACRpedi50 response by month 3 of treatment—this endpoint was achieved in 78/100 (78%) patients. The secondary endpoint (achieving inactive disease by month 6 of therapy) was reached in 48/93 (52%) patients. Given the refractory nature of the disease in this cohort—resistant to multiple lines of therapy including biologic DMARDs with distinct mechanisms of action—these results are highly encouraging. During RTX treatment, arthritis activity decreased and systemic manifestations resolved as early as 3 months post-infusion. This marked clinical improvement was accompanied by normalization of inflammatory markers, including ESR and serum CRP levels, which reached reference values by month 3 post-initiation. One of the goals of disease activity control is reducing the dose and frequency of glucocorticoids (GCs). In our cohort, RTX demonstrated a clear steroid-sparing effect: during year 1 of therapy, the median daily oral prednisolone-equivalent dose was halved and reached its minimum by year 4 of follow-up. During year 1 of RTX therapy, additional intravenous and intra-articular GC administrations were successfully avoided.

Another objective of this study was to evaluate the safety profile of RTX in patients with JIA. According to published literature, serious adverse events occur in 1–3% of patients treated with RTX, with the majority recorded during the first 6 months of therapy [21]. Infusion-related reactions have been reported in 10% to 80% of all RTX infusions, with serious infusion reactions occurring in up to 10% of cases—findings that align with our data [22].

The most extensive safety data on RTX were published by van Vollenhoven et al., spanning an 11-year follow-up period (14,816 patient-years). The incidence rate of serious adverse events (SAEs) in that study was 3.76 per 100 PY. In our study, the SAE rate was 0.8 per 100 PY, which was substantially lower than that reported by van Vollenhoven et al. The majority of AEs were recorded during the first year of therapy; however, in the adult cohort, cardiovascular events constituted the largest proportion of AEs [23]. Among infectious AEs, Roland F. van Vollenhoven et al. reported upper respiratory tract infections (19%), urinary tract infections (11%), and bronchitis (10%) as the most frequent. According to our study data, the most common were pneumonia (29%), ENT infections (20.5%), and urinary tract infections (15%).

Safety data on RTX treatment from the national registry (GRAD) assesses infection episodes in autoimmune disease patients who received RTX therapy. Clinically significant infections mostly occurred within seven months after the first infusion of rituximab. Most infections were mild (3.8%) or moderate (32%), and 3.6% of patients had severe infections [21].

In the study by Russel C. Dale et al. assessing the efficacy and safety of rituximab in 144 patients with central nervous system autoimmune diseases, 138 patients were followed for more than 6 months [24]. According to the authors, the most frequent serious adverse events were infusion reactions (life-threatening in 2% of cases); two patients (1.4%) died due to infectious complications; and seven serious infectious adverse events were recorded during the study.

In the study by Kamei K. et al., the development of agranulocytosis was described in 11/114 (9.6%) patients, occurring on average 66 days after rituximab administration [25]. In our cohort, neutropenia was observed in one-third of patients, and agranulocytosis in 16/106 (15.1%) patients.

A total of 207 patients, with a median age of 12 years, participated in the study conducted by Labrosse et al. Following RTX treatment, there was a statistically significant increase in hypogammaglobulinemia compared to baseline measurements. The prevalence of hypo-IgG significantly rose from 28.7% to 42.6% (*p* = 0.009), hypo-IgA from 11.1% to 20.4% (*p* = 0.02), and hypo-IgM from 20.0% to 62.0% (*p* < 0.0001). Low IgG levels at any time following RTX therapy were associated with a higher risk of severe infections, with an incidence of 34.4% compared to 18.9% (odds ratio, 2.3; 95% confidence interval, 1.1–4.8; *p* = 0.03). Among 101 evaluable patients, 27 (26.7%) exhibited persistent IgG hypogammaglobulinemia. These findings underscore the importance of comprehensive immunologic monitoring both prior to and following rituximab therapy [24].

Depletion of B cells following rituximab administration leads to disrupted post-vaccination seroconversion. The time preceding the RTX infusion is critical. In the initial six to twelve months post-rituximab infusion, post-vaccination antibody levels significantly decrease. The persistence of antibody levels in the context of RTX administration is associated with the presence of residual B cells capable of producing antibodies prior to complete CD20 depletion [25,26]. The reduction in the immune response to vaccines after RTX is more pronounced with polysaccharide and neoantigen vaccines, while T-dependent responses to protein antigens may remain relatively intact, indicating some residual function of plasma cells. Vaccines should be administered at least four weeks before starting RTX. Cellular and partially humoral responses are preserved, so some vaccines, such as annual flu vaccines, can be administered during RTX use [27].

Rituximab increases the risk of developing infections by causing hypogammaglobulinemia and neutropenia, especially with repeated courses of treatment [28].

Numerous risk factors have been associated with the development of hypogammaglobulinemia following RTX, such as a low baseline serum Ig level, the number of RTX courses, and concomitant immunosuppressive drugs [29,30]. Low IgG levels have been registered in 15–40% of adult patients treated with RTX and in up to 30% of patients in pediatric cohorts; a low IgG level seems to be a risk factor for severe infection development, but a low IgM level does not increase risk [27]. In a cohort study of 4479 patients receiving RTX treatment, 1241 of whom had rheumatic disorders, there was a significant increase in the number of severe infections among the overall study population. Increased mortality was associated with severe infections within six months following RTX administration, and higher doses of immunoglobulin therapy were linked to a lower risk of severe infection. Among the rheumatic disease group, 28.2% had severe infections that required hospitalization within 18 months of RTX initiation, with most occurring within the first six months of treatment (*n* = 972) [31]. In patients with recurrent infections and hypogammaglobulinemia, both antibiotic prophylaxis and/or immunoglobulin replacement therapy should be considered; these reduced the incidence and severity of infections [32]. Vaccination is an effective infection prophylaxis but in the case of our study, most of the children were vaccinated according to the National Immunization Schedule and received RTX for life-saving indications, so we suggest blood IgG level correction (passive immunization). The threshold level of IgG at which intravenous immunoglobulin (IVIG) administration should be prophylactically initiated has not been formally determined. Nevertheless, based on guidelines for hematological malignancies, a threshold of less than 400 mg/dL is generally accepted [33].

In conclusion, the results of this four-year observational study demonstrate the efficacy and acceptable safety profile of RTX in children with refractory courses of various juvenile arthritis subtypes, resistant to prior therapy with standard DMARDs and bDMARDs with distinct mechanisms of action. These findings suggest that B-cell-depleting therapy represents a viable therapeutic option for disease control in patients with refractory JIA.

### Study Limitations

The main limitations of this study are related to its retrospective design, the small number of patients in every group, heterogenous study population, partial data loss, the absence of a unified treatment protocol specifying standardized assessment intervals and the number of RTX infusions, and the lack of strict criteria for administering repeat rituximab courses. The decision to administer each repeated treatment course was made on an individual basis (with data collected retrospectively from medical records), and concomitant therapies were not standardized. The patients receiving RTX belonged to a “difficult-to-treat” category; therefore, findings from this study cannot be generalized to the entire population of children with JIA. According to JIA treatment recommendations, RTX is not a first-line agent for this group of diseases and is prescribed off-label, which explains the limited number of observations accumulated over a long period. Patients with RF-positive polyarthritis typically develop the disease in late childhood and are often lost to follow-up by pediatric rheumatologists upon transitioning to adult care at age 18, complicating the assessment of long-term outcomes. The reliability of autoantibody titer changes is also questionable due to the small number of observations at specified time points. We did not perform a subgroup analysis based on JIA subtypes due to the resulting small sample sizes. Indications for RTX initiation were not uniform among patients (ranging from active arthritis to systemic manifestations and laboratory abnormalities). The long data collection period (2005–2025) resulted in a diverse spectrum of medications used both prior to and concomitantly with RTX, complicating adjustments for the impact of prior and concomitant therapies on study outcomes. The missed data introduced bias, which is particularly problematic. Smaller groups and minor variations in participant numbers can lead to inflated or misleading statistical significance. The additional analysis, which includes subgroup analysis as a confounding variable, may alter the study results. These questionable methodological and statistical choices weaken the credibility of the findings. An insufficient sample size, combined with the absence of an initial calculation of the sample size, elevates the risk of Type I error (false positive) and leads to exaggerated claims of the effect. In our study, we did not evaluate the confounding variables and post hoc analysis. These factors could skew the results and interpretations. A more comprehensive analysis that accounts for these confounders would provide more robust and reliable findings. The above-mentioned methodological and statistical shortcomings severely undermine its validity and generalizability.

## 4. Patients and Methods

This retrospective cohort study included 106 patients with JIA treated with RTX at the Department of Pediatric Rheumatology, National Medical Research Center for Children’s Health (Moscow, Russian Federation), between 1 May 2005 and 1 January 2025. Inclusion criteria were a diagnosis of JIA established according to ILAR criteria and documented treatment with RTX [17]. Exclusion criteria were the absence of follow-up medical data or presence of other rheumatic diseases. During the observation period, several patients withdrew from the study. Withdrawal criteria were: absence of medical data or a physician’s decision to change bDMARD. Detailed information regarding the withdrawn patients is provided in Table 5.

Patients received RTX intravenously via two administration regimens: 375 mg/m^2^ of body surface area per dose in four weekly infusions, or 1000 mg per dose in two infusions given two weeks apart. The choice of administration regimen depended on the patient’s height and weight. RTX courses were repeated if high disease activity persisted and/or B-cell depletion was not achieved.

For each patient, we evaluated the following parameters:

- Demographic data: sex, age at disease onset, time from disease onset to RTX administration, and JIA subtype.
- Clinical data: number of joints with active arthritis, number of joints with limited range of motion, morning stiffness duration (in minutes), presence of uveitis, fever, rash, lymphadenopathy, hepatosplenomegaly, serositis, number of systemic features, and presence of macrophage activation syndrome according to EULAR/ACR/PRINTO criteria [34]. Disease activity was assessed using the following parameters: Childhood Health Assessment Questionnaire (CHAQ) [35], Visual Analog Scales (VAS) assessed by parents (pVAS) and physicians (MDVAS), achievement of remission according to C. Wallace criteria [36], and ACRpedi response dynamics [36].
- Laboratory data: hemoglobin level, white blood cell and platelet counts, C-reactive protein (CRP) level, erythrocyte sedimentation rate (ESR), rheumatoid factor (RF), anti-cyclic citrullinated peptide (ACCP) antibodies, IgG levels, and B-cell count. An elevated RF level was defined as >20 IU/L, and an elevated ACCP level was defined as >17 IU/L. B-cell depletion was defined as a CD20-positive B-cell count of <1% of circulating lymphocytes.
- Treatment data: all previous and current treatments and their changes during the study.
- Safety data: evaluated by recording all adverse events during the study and categorizing them according to the Common Terminology Criteria for Adverse Events (CTCAE) v5.0 (published 27 November 2017) [37].

During the 4-year follow-up period, disease activity was measured at baseline (the first RTX infusion) and at 3, 6, 12, 24, 36, and 48 months post-infusion or at withdrawal. The efficacy of RTX was evaluated using the ACRpedi response criteria, C. Wallace inactive disease criteria, and the JADAS71 disease activity index [36,38,39]. Primary and secondary endpoints were established at 3 and 6 months post-initial RTX infusion in accordance with the JIA treat-to-target strategy: the primary endpoint was an ACRpedi50 response at month 3, and the secondary endpoint was the achievement of inactive disease status according to C. Wallace criteria at month 6 [4].

### 4.1. Ethical Considerations

All data were anonymized. This retrospective study was approved by the local ethics committee. RTX therapy was approved by the medical board of the National Medical Research Center for Children’s Health, and informed consent was obtained from all parents and patients older than 14 years of age.

### 4.2. Statistical Analysis

Statistical data were analyzed using Microsoft Excel and RStudio (version 4.2.1). Continuous (quantitative) variables were summarized using the median (Me), interquartile range (IQR), minimum and maximum values [min; max], mean, and standard deviation (SD). Categorical (qualitative) variables were expressed as absolute numbers and percentages. The Shapiro–Wilk test was used to assess data normality. Spearman’s rank correlation coefficient was used to evaluate correlations between continuous variables. To evaluate differences between continuous variables, the Wilcoxon signed-rank test or Student’s *t*-test (for normally distributed paired samples) was used. Due to the withdrawal of patients at every timepoint, the calculations were made according to the actual number of participants. Survival analysis with Kaplan–Meier curves and the Cox proportional hazards model was performed to estimate the probability of achieving the event JIA remission. The missing data were excluded from the analysis. Differences between groups were considered statistically significant at *p* < 0.05.

## 5. Conclusions

The long-term experience with rituximab analyzed in our study suggests that this agent can be considered an additional therapeutic option for children with refractory JIA. Some patients received RTX prior to the era of highly effective targeted therapies for systemic JIA (such as tocilizumab and canakinumab) and non-systemic JIA subtypes (primarily TNF inhibitors). Its use led to a reduction in disease activity, with an ACRpedi50 response achieved in 78% of patients by month 3 of therapy and inactive disease status attained in a subset of patients (52% by month 6). The use of RTX in children with juvenile arthritis appears to be reasonably safe, with 85% of all adverse events reported over the 4-year follow-up period classified as non-severe.

## Figures and Tables

**Table 1:** Demographic characteristics and previous treatment.

Characteristics	Total *n* = 106	
Sex, *n* (%)		
• Male • Female	32 (30) 74 (70)	
Age at disease onset, years, Me (IQR) [min–max]	3.9 (2.3; 8.8) [0.18–16.6]	
Disease duration prior to RTX initiation, years, Me (IQR) [min–max]	3.8 (1.5; 7.2) [0.2–14.4]	
Age at RTX initiation, years, Me (IQR) [min–max]	9.68 (5.4; 14.11) [1.1–17]	
JIA subtype	*n* (%)	
• RF-negative polyarthritis	6 (5.6)	
• Persistent oligoarthritis	2 (2)	
• Extended oligoarthritis	2 (2)	
• Enthesitis-related arthritis	3 (2.8)	
• RF-positive polyarthritis	24 (22.6)	
• Systemic arthritis	69 (65)	
Uveitis, *n* (%)	7 (7)	
History of MAS during disease course, *n* (%)	26 (25)	
**Treatment**	**Patients, *n* (%)**	**Therapy duration, months, Me (IQR) [min–max]**
Oral glucocorticoids	70 (66)	24.3 (9.3; 60.1) [0.25–166]
Intravenous (IV) glucocorticoids	80 (76)	-
Intra-articular (IA) glucocorticoids	40 (38)	-
Methotrexate	99 (93)	27 (8.8; 47) [0.5–164]
Leflunomide	22 (21)	8 (4.2; 30) [0.8–64]
Cyclosporine A	62 (59)	15 (5; 34) [0.5–94.5]
**Other**		
Mycophenolate mofetil Upadacitinib Cyclophosphamide	1 (1) 1 (1) 4 (4)	6 3 -
Previous biologic therapy *	Patients, *n* (%)	
Biologic-naïve	45 (42)	-
Previously treated with 1 biologic agent	39 (37)	-
Previously treated with 2 biologics	14 (13)	-
Previously treated with 3 biologics	5 (5)	-
Previously treated with 4 biologics	2 (2)	-
Previously treated with 5 biologic DMARDs	1 (1)	-
- Infliximab # - Etanercept # - Adalimumab # - Golimumab # - Abatacept # - Tocilizumab # - Canakinumab #	22 (21) 8 (8) 12 (11) 1 (1) 16 (15) 25 (24) 9 (9)	-

* DMARDs: disease-modifying antirheumatic drugs; #: number of patients who received the drug (a single patient may have received different biologic DMARDs sequentially). Abbreviations: IQR, interquartile range; Me, median; min, minimum; max, maximum.

**Table 2:** Baseline characteristics of JIA patients at RTX initiation.

Characteristic	Total *n* = 106	
Joints with limited range of motion, Me (IQR) [min–max]	8 (4; 18) [0–42]	
Active joints, Me (IQR) [min–max]	8 (4; 16) [0–40]	
Morning stiffness duration, minutes, Me (IQR) [min–max]	30 (0; 60) [0–360]	
CHAQ (0–3), score, Me (IQR), [min–max]	1.6 (1.1; 1.9) [0.02–2.9]	
MDVAS (0–100), mm, Me (IQR), [min–max]	42 (35; 46) [19–66]	
pVAS (0–100), mm, Me (IQR), [min–max]	57 (48; 64) [30–89]	
JADAS71, score, Me (IQR), [min–max]	21 (14.8; 30.1) [5–56]	
Systemic manifestations, *n* (%)	62 (58)	
Fever, *n* (%)	19 (18)	
Rash, *n* (%)	58 (55)	
Lymphadenopathy, *n* (%)	25 (24)	
Hepato- and/or splenomegaly, *n* (%)	42 (40)	
Serositis, *n* (%)	9 (8)	
Mean number of systemic manifestations per patient (mean ± SD)	2.22 ± 1.2	
MAS, *n* (%)	6 (6)	
Active uveitis, *n* (%)	3 (3)	
Hemoglobin, g/L, Me (IQR) [min–max]	103 (89; 115) [57–140]	
ESR, mm/h, Me (IQR), [min–max]	43 (25; 56) [20–98]	
CRP, mg/L, Me (IQR), [min–max]	34.5 (11.8; 55) [0–243]	
ACCP, MU/L, Me (IQR), [min, max]	138 (11; 431) [0–1000]	
RF, MU/L, Me (IQR), [min, max]	157 (88; 263) [26–3560]	
**Concomitant therapy**	**Patients, *n* (%)**	**Dose, Ме (IQR) [min–max]**
Oral glucocorticoids, mg/day	57 (54)	10 (5; 18.5) [1.5–55]
IV glucocorticoids	41 (39)	-
IA glucocorticoids	8 (8)	-
Methotrexate, mg/m^2^/week	75 (71)	15 (12; 15) [9–50]
Leflunomide, mg/day	15 (14)	20 (20; 20) [10–20]
Cyclosporine A, mg/kg/day	62 (58)	4 (3.36; 4.49) [0.13–8.3]
Mycophenolate mofetil, mg/m^2^/day	1 (1)	600
Upadacitinib, mg/d	1 (1)	15

Abbreviations: sJIA—systemic juvenile idiopathic arthritis, MAS—macrophage activation syndrome, Me—median, MDVAS—physician-assessed visual analog scale of disease activity, pVAS—parent/patient-assessed visual analog scale of patient global assessment, JADAS71—Juvenile Arthritis Disease Activity Score-71, Hb—hemoglobin, WBC—white blood cells (leucocyte cell count), ESR—erythrocyte sedimentation rate, CRP—C-reactive protein, ACCP—anti-cyclic citrullinated peptide, RF—rheumatoid factor, CHAQ—Childhood Health Assessment Questionnaire.

**Table 3:** Changes in disease activity parameters during RTX treatment.

Characteristics	Baseline (*n* = 106)	Month 3 (*n* = 100)	Month 6 (*n* = 93)	Month 12 (*n* = 76)	Month 24 (*n* = 57)	Month 36 (*n* = 44)	Month 48 (*n* = 31)
Joints with limited range of motion, Me (IQR), [min–max] *p*-value	8 (4; 18) [0–42]	2 (0; 6) [0–31] 0.001 *	2 (0; 6) [0–31] 0.001 *	2 (0; 6) [0–36] 0.001 *	0 (0; 4) [0–40] 0.003 *	0 (0; 4) [0–32] 0.002 *	0 (0; 3) [0–24] 0.001 *
Active joints, Me (IQR), [min–max] *p*-value	8 (4; 16) [0–40]	0 (0; 3) [0–31] 0.001 *	0 (0; 4) [0–18] 0.001 *	0 (0; 3) [0–31] 0.11	0 (0; 1) [0–38] 0.005 *	0 (0; 1) [0–26] 0.001 *	0 (0; 0) [0–14] 0.001 *
Morning stiffness duration, minutes, Me (IQR), [min–max] *p*-value	30 (0; 60) [0–360]	0 (0; 0) [0–240] 0.001 *	0 (0; 0) [0–180] 0.001 *	0 (0; 0) [0–180] 0.001 *	0 (0; 0) [0–120] 0.006 *	0 (0; 0) [0–140] 0.003 *	0 (0; 0) [0–60] 0.04 *
CHAQ disability score (0–3), Me (IQR), [min–max] *p*-value	1.6 (1.1; 1.9) [0.02–2.88]	0.08 (0.02; 0.4) [0.01–2.84] 0.001 *	0.05 (0.02; 0.44) [0.01–2.3] 0.001 *	0.08 (0.02; 0.45) [0.01–2.1] 0.001 *	0.02 (0.02; 0.2) [0.01–2.2] 0.001 *	0.02 (0.01; 0.26) [0.01–2.3] 0.001 *	0.02 (0.01; 0.05) [0.01–2.1] 0.001 *
MDVAS (0–100), mm, Me (IQR), [min–max] *p*-value	42 (35; 46) [19–66]	12 (7; 21) [67–2] 0.001 *	8 (6; 24) [3–56] 0.001 *	8 (6; 24) [3–54] 0.001 *	6 (5; 18) [2–44] 0.001 *	6 (4; 21) [0–56] 0.001 *	5 (4; 8) [0–44] 0.001 *
pVAS (0–100), mm, Me (IQR), [min–max] *p*-value	57 (48; 64) [30–89]	18 (9; 34) [4–90] 0.001 *	14 (8; 38) [4–78] 0.001 *	13 (8; 35) [4–78] 0.001 *	8 (7; 28) [4–68] 0.001 *	11 (6; 32) [0–77] 0.001 *	7 (6; 12) [0–56] 0.001 *
JADAS71 score, Me (IQR), [min–max] *p*-value	21 (14.8; 30.1) [5–56]	2.7 (0; 8.9) [0–49] 0.003 *	1 (0; 11.5) [0–31.9] 0.002 *	1.5 (0; 8.4) [0–43.5] 0.004 *	0 (0; 5) [0–48.4] 0.006 *	1 (0; 6.1) [0–39.7] 0.007 *	0 (0; 1) [0–19] 0.004 *
Hb, g/L, Me (IQR), [min–max] *p*-value	103 (89; 115) [57–140]	112 (101; 120) [62–146] 1	113 (102; 123) [74–149] 0.05	120 (103; 127) [86–153] 0.15	123 (106; 132) [62–147] 0.035 *	120 (115; 133) [94–152] 0.006 *	125 (116; 131) [96–155] 0.003 *
ESR, mm/h, Me (IQR), [min–max] *p*-value	43 (25; 56) [20–98]	20 (20; 30) [20–90] 0.001 *	20 (20; 25) [20–73] 0.001 *	20 (20; 20) [20–65] 0.001 *	20 (20; 20) [20–67] 0.001 *	20 (20; 20) [20–55] 0.001 *	20 (20; 20) [20–97] 0.001 *
CRP, mg/L, Me (IQR), [min–max] *p*-value	34.5 (11.8; 55) [0–243]	0 (0; 16.75) [0–193] 0.03 *	0 (0; 11) [0–121] 0.04 *	0 (0; 10) [0–120] 0.03 *	0 (0; 12) [0–93] 0.03 *	0 (0; 11) [0–128] 0.04 *	0 (0; 0) [0–29] 0.04 *
IgG, g/L, Me (IQR), [min–max] *p*-value *	11.3 (9.8; 13.5) [3.2–23.6]	9.4 (7.5; 11.8) [2.3–20.9] 0.05	8.9 (7.3; 11.64) [1.37–20.9] 0.01 *	9 (6.6; 11.0) [1.1–15] 0.01 *	8.2 (6.3; 10.74) [0.87–16.7] 0.03 *	6.8 (5.1; 9.2) [1.1–14.8] 0.02 *	7.9 (6; 10.2) [0.44–23.8] 0.03 *
Patients with B-cell depletion, *n* (%)	–	95 (95)	75 (81)	36 (47)	22 (39)	17 (39)	8 (31)
Patients with systemic features, *n* (%)	62 (58)	15 (15)	11 (12)	7 (9)	6 (11)	3 (7)	0 (0)
Number of systemic features per case, mean ± SD	2.22 ± 1.2	0.39 ± 0.8	0.31 ± 0.69	0.2 ± 0.49	0.18 ± 0.65	0.12 ± 0.42	0 ± 0
Patients with RF-positive arthritis, *n*	24 (23)	20 (20)	18 (19)	12 (16)	5 (9)	2 (5)	2 (6)
ACCP, IU/L, Me (IQR), [min–max] *p*-value	138 (11; 431) [0–1000]	55 (7; 195) [0–679] 0.03 *	100 (4; 267) [0–560] –	180 (40; 369) [0–1500] –	30 (7; 500) [0–780] –	200 (100; 310) [0–420] –	589 (545; 634) [500–678] –
RF, IU/L, Me (IQR), [min–max] *p*-value	157 (88; 263) [26–3560]	50 (24; 64) [0–890] 0.002 *	55 (25; 83) [0–1000] 0.02 *	42 (27; 91) [0–655] 0.03 *	30 (20; 59) [0–1500] –	56 (28; 186) [0–316] –	986 (522; 1447) [60–1910] –

Me—median, MDVAS—physician-assessed visual analog scale of disease activity, pVAS—parent/patient-assessed visual analog scale of patient global assessment, JADAS71—Juvenile Arthritis Disease Activity Score-71, Hb—hemoglobin, ESR—erythrocyte sedimentation rate, CRP—C-reactive protein, ACCP—anti-cyclic citrullinated peptide, RF—rheumatoid factor, CHAQ—Childhood Health Assessment Questionnaire, * *p*-value < 0.05 statistically significant.

**Table 4:** Adverse events during rituximab therapy.

Adverse Events	Year 1 (*n* = 203)	Year 2 (*n* = 71)	Year 3 (*n* = 55)	Year 4 (*n* = 13)	Total (*n* = 347)
Infusion-related reactions (IRRs, total)	66	20	12	-	98
Nausea/vomiting, *n*	10	-	-	-	10
Skin rash, *n*	19	6	4	-	29
Abdominal pain, *n*	8	3	-	-	11
Headache, *n*	8	2	-	-	10
Dyspnea, *n*	12	5	2	-	19
Flu-like syndrome, *n*	37	7	4	-	48
Sore throat, *n*	6	5	4	-	15
Facial and mucosal edema, *n*	2	1	-	-	3
Anaphylaxis, *n*	2	-	-	-	2
IRR (grade < 3)	54	16	11	0	81
IRR (grade 3)	11	4	1	0	16
IRR (grade > 3)	1	0	0	0	1
Infectious adverse events (total)	69	28	27	10	134
Pneumonia, *n*	23	9	8	1	41
ENT, *n*	11	8	4	2	25
Herpes viral infections, *n*	6	1	1	0	8
Mucosal fungal infections, *n*	1	1	2	0	4
Acute intestinal infections (AIIs), *n*	7	1	1	1	10
UTIs, *n*	9	2	7	3	21
Skin and soft tissue infections, *n*	4	3	0	0	7
ARI, *n*	5	3	3	2	13
Septic arthritis of the knee (purulent gonitis), *n*	0	0	0	1	1
Sepsis, *n*	2	0	1	0	3
Tuberculosis, *n*	1	0	0	0	1
Infectious adverse events (grade <3)	54	24	23	9	110
Infectious adverse events (grade 3)	14	4	4	1	23
Infectious adverse events (grade >3)	1 *	0	0	0	1
Other adverse events (total)	71	25	16	3	115
Agranulocytosis, *n*	14	2	2	0	18
Anemia, *n*	1	1	0	0	2
Thrombocytopenia, *n*	1	0	0	0	1
Elevated liver enzymes, *n*	6	4	1	0	11
Elevated blood urea nitrogen/urea, *n*	1	0	0	0	1
Hypogammaglobinemia (low Ig levels), *n*	45	16	13	3	77
MAS, *n*	3	0	0	0	3
Active uveitis, *n*	0	2	0	0	2
Other adverse events (grade < 3)	64	21	16	3	104
Other adverse events (grade = 3)	4	4	0	0	8
Other adverse events (grade > 3)	3	0	0	0	3

Abbreviations: IRRs—infusion-related reactions; ARIs—acute respiratory infections; ENT—ear, nose, and throat infections; AIIs—acute intestinal infections; UTIs—urinary tract infections; MAS macrophage activation syndrome, * *p*-value < 0.05 statistically significant.

**Table 5:** Patient withdrawals during the observation period.

Time Since 1st RTX Administration	Patients Followed Up (*n*)	Withdrawn Patients (*n*)	Causes of Withdrawal * and Number of Excluded Patients
3 months	100	6	• MAS (switched to another biologic DMARD), *n* = 2; • Death due to SARS-CoV-2, *n* = 1; • Transition to adult rheumatology clinics with loss of follow-up data, *n* = 3.
6 months	93	7	• Switched to other biologic DMARDs, *n* = 2 (persistent arthritis, *n* = 1; unavailability of RTX, *n* = 1); • MAS (switched to other biological DMARDs), *n* = 1; • Transition to Adult Rheumatology Clinics with loss of the following data, *n* = 4.
12 months	76	17	• Switched to other biological DMARDs, *n* = 13, including - Switched to TNFi, *n* = 7 (JIA flare, *n* = 4; lack of RTX efficacy, *n* = 3); - Switched to tocilizumab due to secondary loss of efficacy, *n* = 3; - Switched to canakinumab due to persistent systemic manifestations, *n* = 3. - Tuberculosis, *n* = 1. - No data available, *n* = 3.
24 months	57	19	• Switched to other biological DMARDs, *n* = 4 (recurrent infections, *n* = 1; lack of efficiency, *n* = 3); • Active uveitis (switched to another biologic DMARD), *n* = 2; • Transition to Adult Rheumatology Clinics with loss of the following data, *n* = 13.
36 months	44	13	• Switched to other biologic DMARDs, *n* = 9 (recurrence of systemic symptoms in systemic JIA, *n* = 4; lack of efficacy, *n* = 5); • Transition to Adult Rheumatology Clinics with loss of the following data, *n* = 4; • No available data, *n* = 1.
48 months	31	13	• Switched to other biologic DMARDs, *n* = 7 (infections, *n* = 2; systemic manifestations, *n* = 2; persistent arthritis, *n* = 5); • Transition to Adult Rheumatology Clinics with loss of the following data, *n* = 4; • No available data, *n* = 2.

* During the previous observation period. DMARDs—disease-modifying antirheumatic drugs; MAS—macrophage activation syndrome; SARS-CoV-2—severe acute respiratory syndrome coronavirus 2; RTX—rituximab.

## Data Availability

The datasets generated during and/or analyzed during the current study are available from the corresponding author upon reasonable request and subject to institutional approval. The data are not publicly available due to ethical and privacy restrictions related to patient confidentiality.
